# Evaluation of Lymph Node Metastasis in Advanced Gastric Cancer Using Magnetic Resonance Imaging-Based Radiomics

**DOI:** 10.3389/fonc.2019.01265

**Published:** 2019-11-22

**Authors:** Wujie Chen, Siwen Wang, Di Dong, Xuning Gao, Kefeng Zhou, Jiaying Li, Bin Lv, Hailin Li, Xiangjun Wu, Mengjie Fang, Jie Tian, Maosheng Xu

**Affiliations:** ^1^First College of Clinical Medicine, Zhejiang Chinese Medical University, Hangzhou, China; ^2^Department of Radiology, The First Affiliated Hospital of Zhejiang Chinese Medical University, Hangzhou, China; ^3^Department of Gastroenterology, The First Affiliated Hospital of Zhejiang Chinese Medical University, Hangzhou, China; ^4^CAS Key Laboratory of Molecular Imaging, Institute of Automation, Chinese Academy of Sciences, Beijing, China; ^5^School of Artificial Intelligence, University of Chinese Academy of Sciences, Beijing, China; ^6^Beijing Advanced Innovation Center for Big Data-Based Precision Medicine, School of Medicine, Beihang University, Beijing, China

**Keywords:** lymph node metastasis, magnetic resonance imaging, diffusion-weighted imaging, advanced gastric cancer, radiomics

## Abstract

**Objective:** To develop and evaluate a diffusion-weighted imaging (DWI)-based radiomic nomogram for lymph node metastasis (LNM) prediction in advanced gastric cancer (AGC) patients.

**Overall Study:** This retrospective study was conducted with 146 consecutively included pathologically confirmed AGC patients from two centers. All patients underwent preoperative 3.0 T magnetic resonance imaging (MRI) examination. The dataset was allocated to a training cohort (*n* = 71) and an internal validation cohort (*n* = 47) from one center along with an external validation cohort (*n* = 28) from another. A summary of 1,305 radiomic features were extracted per patient. The least absolute shrinkage and selection operator (LASSO) logistic regression and learning vector quantization (LVQ) methods with cross-validations were adopted to select significant features in a radiomic signature. Combining the radiomic signature and independent clinical factors, a radiomic nomogram was established. The MRI-reported N staging and the MRI-derived model were built for comparison. Model performance was evaluated considering receiver operating characteristic (ROC) analysis, calibration curves, and decision curve analysis (DCA).

**Results:** A two-feature radiomic signature was found significantly associated with LNM (*p* < 0.01, training and internal validation cohorts). A radiomic nomogram was established by incorporating the clinical minimum apparent diffusion coefficient (ADC) and MRI-reported N staging. The radiomic nomogram showed a favorable classification ability with an area under ROC curve of 0.850 [95% confidence interval (CI), 0.758–0.942] in the training cohort, which was then confirmed with an AUC of 0.857 (95% CI, 0.714–1.000) in internal validation cohort and 0.878 (95% CI, 0.696–1.000) in external validation cohort. Meanwhile, the specificity, sensitivity, and accuracy were 0.846, 0.853, and 0.851 in internal validation cohort, and 0.714, 0.952, and 0.893 in external validation cohort, compensating for the MRI-reported N staging and MRI-derived model. DCA demonstrated good clinical use of radiomic nomogram.

**Conclusions:** This study put forward a DWI-based radiomic nomogram incorporating the radiomic signature, minimum ADC, and MRI-reported N staging for individualized preoperative detection of LNM in patients with AGC.

## Introduction

Gastric cancer is a common and debilitating disease negatively impacting the physical and mental health of patients, worldwide. The onset of early gastric cancer is concealed, and most of them have become advanced gastric cancer (AGC) related to poor prognosis when clinically discovered (1). Evidence from studies shows that perioperative treatment of AGC (neoadjuvant chemotherapy/radiotherapy and adjuvant chemotherapy/radiotherapy) has been proven superior to surgery alone in many Western countries. The Chinese Society of Clinical Oncology also indicates that preoperative chemotherapy can well-improve the tumor remission rate and R0 resection rate with good safety in Asian countries based on D2 lymphadenectomy studies (2). As a crucial factor affecting the prognosis quality and survival of AGC patients, knowing the lymph node metastasis (LNM) status in advance has potential guiding significance for the decision making of therapeutic strategies including neoadjuvant chemotherapy, surgery, or intraoperative lymph node dissection (1–3). Morphological changes of lymph node architecture have been regarded as the reasonable and clinically acknowledged criteria for the determination of LNM currently (3). However, these changes do not correspond exactly to pathology. For example, small lymph nodes have metastasized, while large lymph nodes may be simply caused by inflammation (2, 4). Both errors offer a glimpse into the potential pitfalls of current LNM analysis methods. Therefore, a method allowing more accurate identification of LNM status should be considered as an urgent issue for clinical decision making.

Diffusion-weighted imaging (DWI) describes a magnetic resonance imaging (MRI) sequence which analyzes the Brownian movement of water molecules *in vivo* to determine morphological and functional parameters (5). Currently, DWI is a powerful modality to differentiate malignant and benign legions with the assumption that malignant lesions generally display higher cellularity. However, the correlation between DWI signal and LNM is not completely uniform, so the current accuracy of DWI-based analysis still falls below the clinical requirement in most cases (6).

Radiomics is a burgeoning field which involves converting imaging data into potential high-dimensional radiomic features through a large series of automatic feature extraction and data characterization algorithms (7–9). Quantitative radiomic feature analysis is now a widely recognized method in capturing distinct phenotypic differences along with changes in internal structure from a microscopic perspective (10). An increasing number of high-quality datasets and advanced pattern recognition algorithms have contributed to the rapid growth and development of radiomics (11). Furthermore, previous studies (5, 6, 9, 12) have indicated that certain quantitative radiomic signature had a surprising correlation with the prediction and evaluation of cancers. However, there is no article about DWI-based radiomic models for LNM prediction in AGC yet. Thereby, a combination of radiomics and DWI may provide a reliable method of precision medicine for the individualized prediction of LNM in patients with AGC.

## Overall Study

### Research Materials

#### Patients

Ethical approval for this retrospective study was granted by the ethics committee of the First Affiliated Hospital of Zhejiang Chinese Medical University and Hangzhou Hospital of Traditional Chinese Medicine. We waived the requirement for informed consent. This study consecutively enrolled 146 pathologically diagnosed AGC patients with total or partial radical gastrectomy from February 2016 to December 2018. Supplementary Figure 1 shows the detailed recruitment diagram for study population from the two centers. The inclusion and exclusion criteria are defined as follows.

The inclusion criteria were the following: (a) patients with confirmed AGC according to the American Joint Committee on Cancer staging manual (1), and (b) a standard 3.0 T MRI was performed <2 weeks before surgical resection.

The exclusion criteria were the following: (a) patients with combined malignant neoplasm, distant metastasis, or preoperative therapy (radiotherapy, chemotherapy, or chemoradiotherapy); (b) incomplete clinical information or pathological information; (c) inflammatory diseases, including infections, ischemic heart disease, hereditary gastric cancer, collagen disease, and bowel perforation or obstruction; (d) the total number of intraoperative lymph node dissections was <16; and (e) low MRI resolution or small tumor lesion (<1 cm).

#### MRI Acquisition and Tumor Segmentation

All patients were given written informed consent before MRI examinations. Patients attending the inspection fasted for at least 8 h and drank 700–1,000 ml warm water within 5 min to fill the stomach cavity. Each patient was asked to cooperate with the respiratory training before examination to ensure the normal inspection operation and reduce motion artifacts. A full diagnostic abdominal MRI protocol was executed. The MRI scans, covering the entire stomach region from the diaphragmatic dome to the level of the renal hilum, were performed during a breath-hold, with the patient supine in all the phases. All the patients underwent MRI scans successfully without any discomfort.

The images were exported from the Institutional Picture Archiving and Communication System (PACS, Carestream). MRI was performed using a whole-body 3.0 T scanner (Discovery 750, GE Healthcare, Milwaukee, WI, USA). Eight-channel head phased array coils and conventional sequences were used to obtain all the sequences. The scanning parameters of Axial DWI Shim are as follows: gradient factor *b* values are 0, 1,000 s/mm^2^, matrix 128 × 130, TE = minimum, number of layers are 26 (maximum slices are 38), thickness of layer is 6.0 mm, spacing between layers is 2.0 mm, NEX for T2 is 4.00.

Manual segmentation of the entire tumor volume of interest (VOI) was conducted with ITK-SNAP software (version 3.6; www.itksnap.org) on the axial DWI sequence. VOI included the inner border of the lesion on whole axial slices and avoided necrotic tissue and surrounding adipose tissue (5). The T2-weighted images and contrast-enhanced T1-weighted images were used as references for the VOI segmentation on DWI sequence.

Three-dimensional volume images were delineated by two radiologists (WC and XG, with 7 and 25 years of experience in MRI abdominal diagnosis, respectively). They were both blind to pathological information of patients. WC performed tumor segmentation for all 146 patients and then repeated the segmentation procedure after 2 weeks on 30 randomly selected patients to test the intrareader consistency. XG only segmented the above 30 cases to assess the interreader consistency of the radiomic features.

#### Clinical Factors

Clinical factors for center 1 patients in this study are summarized in Table 1, including age, sex, the primary site of the tumor, tumor size, MRI-reported T staging, MRI-reported N staging, pathological T staging, average apparent diffusion coefficient (ADC) value, minimum ADC value, and combined markers (CA19-9, CA72-4, and CEA). The clinical factors for center 2 patients are given in Supplementary Table 1. The detailed grouping criteria are given as follows.

**Table 1:** Clinical and imaging characteristics of patients with AGC.

**Clinical factors**	**Training cohort (*****n*** **=** **71)**		***p* value**	**Validation cohort (*****n*** **=** **47)**		***p* value**
	**LNM (+)**	**LNM (–)**		**LNM (+)**	**LNM (–)**	
Age, mean ± SD, years	64.7 ± 12.22	66.06 ± 11.06	0.7458	61.24 ± 13.67	67.77 ± 7.51	0.1566
Sex, no. (%)			0.1615			0.6921
Male	43 (81.1)	11 (61.1)		28 (82.4)	10 (76.9)	
Female	10 (18.9)	7 (38.9)		6 (17.6)	3 (23.1)	
Primary site, no. (%)			0.5205			0.1796
Upper	12 (22.6)	6 (33.3)		6 (17.6)	3 (23.1)	
Middle	19 (35.8)	7 (38.9)		15 (44.1)	2 (15.4)	
Under	22 (41.5)	5 (27.8)		13 (38.2)	8 (61.5)	
Tumor size, no. (%)			0.1134			0.6771
<5.0 cm	21 (39.6)	11 (61.1)		15 (44.1)	7 (53.8)	
≥5.0 cm	32 (60.4)	7 (38.9)		19 (55.9)	6 (46.2)	
MRI-reported T staging, no. (%)			0.1726			0.4597
T2–3	17 (32.1)	9 (50.0)		10 (29.4)	6 (46.2)	
T4	36 (67.9)	9 (50.0)		24 (70.6)	7 (53.8)	
MRI-reported N staging, no. (%)			0.0172^*^			0.0489
Positive	42 (79.2)	9 (50.0)		29 (85.3)	7 (53.8)	
Negative	11 (20.8)	9 (50.0)		5 (14.7)	6 (46.2)	
pT staging, no. (%)			0.0005^*^			<0.0001^*^
T2–3	8 (15.1)	11 (61.1)		2 (5.9)	9 (69.2)	
T4	45 (84.9)	7 (38.9)		32 (94.1)	4 (30.8)	
Average ADC value, mean	1,419 (74.6)	1,387 (25.4)	0.9473	1,428 (72.3)	1,499 (27.7)	0.3788
Minimum ADC value, no. (%)			0.0312^*^			0.0095^*^
0 (<700)	7 (13.2)	5 (27.8)		1 (2.9)	4 (30.8)	
1 (700–1,200)	36 (67.9)	6 (33.3)		26 (76.5)	5 (38.4)	
2 (≥1,200)	10 (18.9)	7 (38.9)		7 (20.6)	4 (30.8)	
Combined makers, no. (%)			0.2458			0.4146
Positive	29 (54.7)	7 (38.9)		14 (41.2)	3 (23.1)	
Negative	24 (45.3)	11 (61.1)		20 (58.8)	10 (76.9)	
Radiomic signature			<0.0001^*^			0.0059^*^
Median	1.771	0.215		1.913	1.024	
(Interquartile range)	(1.136–2.495)	(−0.258–0.981)		(1.059–2.685)	(0.251–1.461)	
Radiomic nomogram			<0.0001^*^			<0.0001^*^
Median	2.073	0.245		2.273	0.452	
(Interquartile range)	(1.064–2.989)	(−0.610–0.843)		(1.868–2.892)	(−0.047–0.957)	

p values are calculated from univariate analysis between each clinical factor and corresponding LNM status. AGC, advanced gastric cancer; LNM, lymph node metastasis; MRI, magnetic resonance imaging; pT staging, pathological T staging; ADC, apparent diffusion coefficient; SD, standard deviation;

* *p < 0.05*.

##### MRI-reported N staging

Patients were classified as N-positive if a regional lymph node with a measurement of >8 mm on its shortest axis was found, or if a regional lymph node had a higher signal intensity than muscle. The absence of enlarged (>8 mm) or hyperintense lymph nodes was defined as N-negative, which was consistent with the definition of radiological positive nodal status in most previous studies.

##### Primary site of the tumor

In the coronal position, the stomach was divided into upper, middle, and lower parts according to the tripartite connection of the greater curvature and the lesser curvature.

##### MRI-reported T staging

“T4 staging” defines a tumor lesion that infiltrates the serous layer, while T3 or T2 denotes a tumor that has not invaded the serous layer.

##### Combined markers

A combined marker was defined as positive when either of the three markers (CA19-9, CA72-4, and CEA) was positive, and all maker results came from the examination 1 week before surgery.

### Radiomic Analysis Procedures

**Figure 1:**
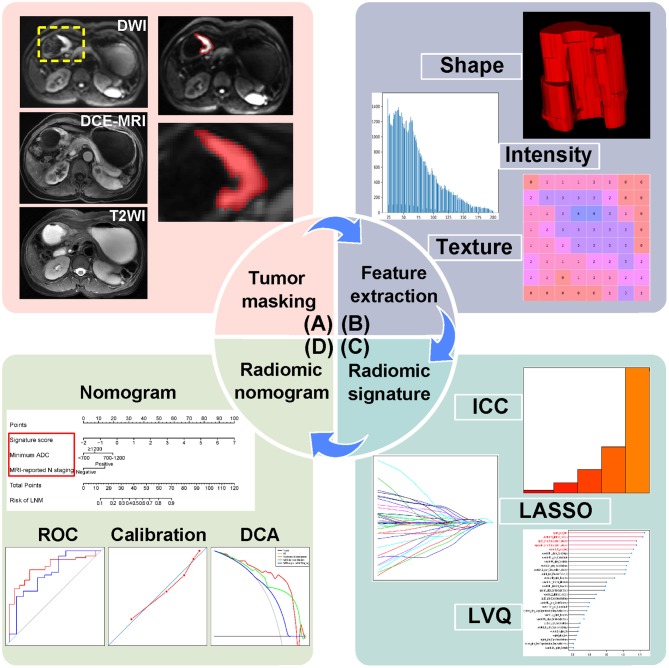
Radiomics workflow of this study. **(A)** Tumor masking of AGC patients based on DWI. **(B)** Radiomic feature extraction, quantifying tumor shape, intensity, and texture. **(C)** Strategies for radiomic signature development. **(D)** Radiomic nomogram with evaluation of ROC, calibration curves, and DCA. AGC, advanced gastric cancer; DWI, diffusion-weighted imaging; DCE-MRI, dynamic contrast enhanced-magnetic resonance imaging; T2WI, T2-weighted images; ICC, intraclass correlation coefficient; LASSO, least absolute shrinkage and selection operator; LVQ, learning vector quantization; ROC, receiver operating characteristic; DCA, decision curve analysis.

A dataset of 118 AGC patients from center 1 were separated into a training cohort (*n* = 71) and an internal validation cohort (*n* = 47) at a ratio of 3:2 randomly. Patients from center 2 constituted an external validation cohort (*n* = 28). As shown in Figure 1, the radiomics workflow consists of four steps, including tumor masking, radiomic feature extraction, radiomic signature construction, and radiomic nomogram development and evaluation.

#### Radiomic Feature Extraction

Radiomic features in this study were extracted from tumor VOIs on DWI images with algorithms implemented in Python 2.7 (https://www.python.org). The radiomic features (summarized in Supplementary Table 2) were composed of three groups: shape features, first-order features, and texture features.

To test the reproducibility and stability of extracted features, intraclass correlation coefficients (ICCs) were calculated (Supplementary Material 1.1). Features with ICC values >0.75 were reserved due to their good reproducibility. Then, all radiomic features were normalized.

#### Feature Selection and Radiomic Signature Construction

Radiomic feature selection as well as radiomic signature construction were carried out in the training cohort. The least absolute shrinkage and selection operator (LASSO) logistic regression was conducted by 5-fold cross-validation for feature reduction. Then, radiomic features were ranked according to their importance to LMN status using learning vector quantization (LVQ). LVQ is a kind of supervised neural network algorithm using a small number of weighted vectors to represent original data based on Euclidean distance measurements (13). Comparative out-of-bag (OOB) bootstrapping estimates with logistic regression models were performed 10 times for each feature subset consisting of the top 5, 10, 15, 20, and 25 features from LVQ, respectively. The average testing area under curve (AUC) and average bias between training AUC and testing AUC from 10 measurements were used as an approach to confirm the number of features in the optimal feature subset. Backward stepwise elimination with Akaike information criterion was then applied. Finally, selected radiomic features weighted by corresponding logistic regression coefficients provided a linear mathematical formula to calculate a radiomic signature.

#### Performance Evaluation of Radiomic Signature

Pearson correlation coefficients were calculated to verify definite contribution of the radiomic signature in classifying LNM status in the training and internal validation cohorts. Receiver operating characteristic (ROC) curves and AUCs were used to evaluate the performance of radiomic signature in the three cohorts. Sensitivity, specificity, and accuracy results were also calculated.

#### Development of Radiomic Nomogram

Preoperative clinical factors shown in Table 1 were taken into consideration to establish a more powerful predictive radiomic nomogram. In univariate analysis for selecting significant clinical factors in the training cohort, Mann-Whitney *U*-test was used for numerical variables, and Chi-square test and fisher's exact test were applied for categorical features. Subsequently, multivariate logistic regression was used to build a radiomic nomogram by integrating radiomic signature and significant clinical factors. The output of the radiomic nomogram is the probability of LNM.

#### Assessment of Radiomic Nomogram

The radiomic nomogram was assessed by ROC curves and AUC values in the training, internal validation, and external validation cohorts. Calibration curves as well as Hosmer-Lemeshow tests were used to assess the fitting degree of radiomic nomogram. An MRI-derived model constructed by significant clinical factors and an MRI-reported N staging scheme was developed for comparison. Sensitivity, specificity, and accuracy results of comparative experiments were also calculated.

Decision curve analysis (DCA) was carried out in the internal validation cohort by quantifying the net benefits at some threshold probabilities and determining clinical use of radiomic nomogram.

#### Statistical Analysis

A two-sided *p* < 0.05 of every statistical test was deemed significantly different, and all analyses were based on R language (version 3.4.3; https://www.r-project.org). R packages used in our work are described in Supplementary Material 1.2.

## Results

### Clinical Factors

Baseline characteristics of patients from center 1 are shown in Table 1. LNM positive patients covered 74.6% (53/71) and 72.3% (34/47) of the training and internal validation cohorts, respectively, with no significant difference (*p* = 0.7804, Chi-square test) in LNM status between the two cohorts. There showed no significant statistical difference in sex (*p* = 0.5384), age (*p* = 0.5039), and all the other clinical factors (*p* = 0.1202–0.7747) between the two cohorts. LNM status had significant associations with MRI-reported N staging (*p* = 0.0172) and minimum ADC (*p* = 0.0312), while other clinical factors were excluded during the univariate analysis.

### Feature Selection and Radiomic Signature Building

**Figure 2:**
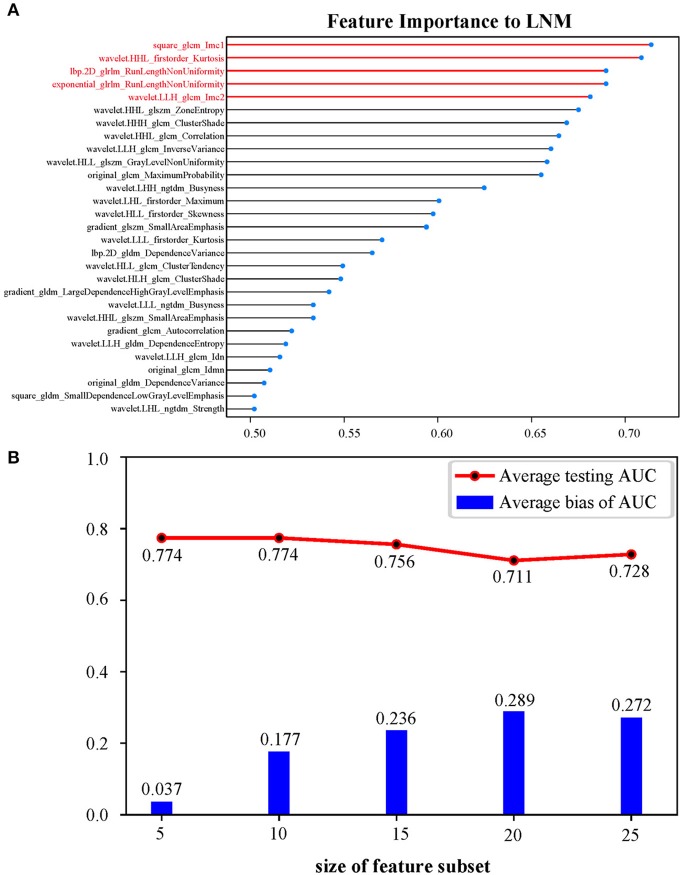
**(A)** Twenty-nine features ranked in descending order of importance to LNM by LVQ. **(B)** OOB bootstrapping estimates with logistic regression of top 5, 10, 15, 20, and 25 features ranked by LVQ, respectively, confirming an optimal feature subset of five features. Average bias of AUC equals average training AUC minus average testing AUC. LNM, lymph node metastasis; LVQ, learning vector quantization; OOB, out-of-bag; AUC, area under the curve.

Among 1,305 original radiomic features per patient, 813 features were first selected after ICC analysis. Then, the multivariate LASSO method indicated 29 potential features (Supplementary Figures 2A,B). As shown in Figure 2, a logistic regression model consisting of features ranking the top 5 in LVQ method gained a higher average testing AUC (0.774) and a smaller average bias between training AUC and testing AUC (0.037). After backward stepwise selection, two key features (*square_glcm_Imc1, p* = 0.0013; *wavelet.LLH_glcm_Imc2, p* = 0.0062) remained and made up the radiomic signature. Detailed explanations for the two radiomic features are given in Supplementary Material 1.3. The formula for the radiomic signature is given as below.

$$\begin{array}{l} \text{Radiomic signature}=-1.3383\times square\text{\_}glcm\\ \text{ \_}Imc1-1.0139\times wavelet.LLH\\ \text{ \_}glcm\text{\_}Imc2+1.5145 \end{array}$$

### The Performance of Radiomic Signature

There was a significant correlation (Pearson's *r* = 0.448, 0.432, and 0.458) between the radiomic signature and LNM status in the three cohorts. A significant difference (*p* < 0.0001) was found in radiomic signature [median (interquartile range)] between LNM and non-LNM groups in training cohort [1.771 (1.136–2.495) vs. 0.215 (−0.258–0.981), respectively]. This difference was confirmed in the validation cohort [1.913 (1.059–2.685) vs. 1.024 (0.251–1.461), *p* = 0.0059]. As estimated, patients with LNM generally got a higher radiomic signature score than those with non-LNM. The distinguishing ability of radiomic signature in training cohort and internal validation cohort was indicated with an AUC of 0.821 [95% confidence interval (CI), 0.720–0.922] and 0.758 (95% CI, 0.591–0.925), respectively. Furthermore, the AUC in external validation cohort achieved 0.741 (95% CI, 0.513–0.971). Detailed sensitivity, specificity, and accuracy results are presented in Table 2. Their corresponding 95% CI are attached in Supplementary Table 3.

**Table 2:** Performance evaluation of models in three cohorts.

**Cohorts**	**Models**	**TP**	**TN**	**FN**	**FP**	**Sensitivity**	**Specificity**	**Accuracy**	**AUC (95% CI)**
**Training**									
	MRI-reported N staging	42	9	11	9	0.792	0.500	0.718	0.646 (0.515–0.777)
	MRI-derived model	28	15	25	3	0.528	0.833	0.606	0.736 (0.602–0.871)
	Radiomic signature	42	14	11	4	0.792	0.778	0.789	0.821 (0.720–0.922)
	Radiomic nomogram	36	17	17	1	0.679	0.944	0.746	0.850 (0.758–0.942)
**Internal validation**									
	MRI-reported N staging	29	6	5	7	0.853	0.462	0.745	0.657 (0.504–0.811)
	MRI-derived model	21	12	13	1	0.618	0.923	0.702	0.818 (0.688–0.948)
	Radiomic signature	26	6	8	7	0.765	0.462	0.681	0.758 (0.591–0.925)
	Radiomic nomogram	29	11	5	2	0.853	0.846	0.851	0.857 (0.714–1.000)
**External validation**									
	MRI-reported N staging	17	5	4	2	0.810	0.714	0.786	0.762 (0.562–0.962)
	MRI-derived model	15	7	6	0	0.714	1.000	0.786	0.884 (0.765–1.000)
	Radiomic signature	20	1	1	4	0.952	0.429	0.821	0.741 (0.513–0.971)
	Radiomic nomogram	20	5	1	2	0.952	0.714	0.893	0.878 (0.696–1.000)

*MRI, magnetic resonance imaging; TP, true positive; TN, true negative; FN, false negative; FP, false positive; AUC, area under the receiver operating characteristic curve; CI, confidence interval*.

Given the limited sample size, a 10-fold cross-validation in the center 1 cohort was conducted to avoid overfitting. Results given in Table 3 indicated an average bias across 10-fold of 0.052 between training AUC values and validation AUC values. In addition, feature selection was conducted in each fold. The histogram in Supplementary Figure 3 summarized the counts of selected feature's appearance, showing that the two radiomic features (*square_glcm_Imc1, wavelet.LLH_glcm_Imc2*) used in our radiomic signature appeared most frequently and were the most stable.

**Table 3:** Ten-fold cross-validation to build radiomic signature in center 1 cohort.

**Index**	**AUC**		**Bias**	**Number of features**
	**Training**	**Validation**		
1	0.841	0.556	0.285	7
2	0.795	0.700	0.095	2
3	0.796	0.833	−0.037	2
4	0.788	1.000	−0.212	2
5	0.802	0.833	−0.031	2
6	0.710	0.722	−0.012	2
7	0.745	0.533	0.212	2
8	0.868	0.611	0.257	5
9	0.866	0.800	0.066	5
10	0.773	0.875	−0.102	2
Average bias	0.798	0.746	0.052	

*Bias equals training AUC value minus validation AUC. Numbers of radiomic features selected in each fold are given. AUC, area under the receiver operating characteristic curve*.

### Development and Assessment of Radiomic Nomogram

**Figure 3:**
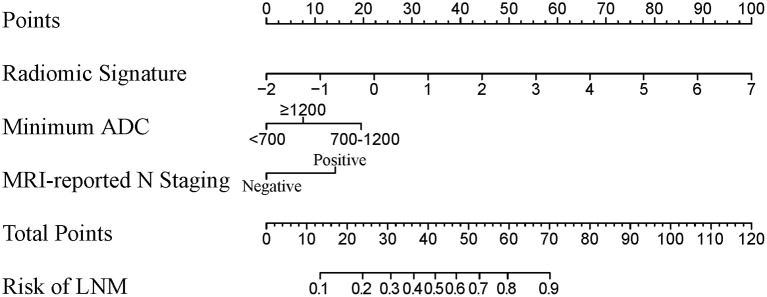
A radiomic nomogram integrated radiomic signature, clinical minimum ADC value, and MRI-reported N staging. The value of each predictor can be converted into a risk score according to the “Points.” After adding up the individual risk score of these predictors in “Total Points,” we can get the corresponding prediction probability of LNM in “Risk of LNM” at the bottom. ADC, apparent diffusion coefficient; MRI, magnetic resonance imaging; LNM, lymph node metastasis.

A radiomic nomogram combining the radiomic signature, minimum ADC value, and MRI-reported N staging is shown in Figure 3. The formula for the radiomic nomogram is shown as below, where “*IF(minimum ADC* = *1)*” represents 700 ≤ minimum ADC < 1,200, “*IF(minimum ADC* = *2)*” means minimum ADC ≥ 1,200, and “*IF(MRI-reported N staging* = *1)*” represents positive MRI-reported N staging. MRI-derived model was built by minimum ADC and MRI-reported N staging.

$$\begin{array}{l} \text{Radiomic nomogram }=\text{0}.\text{8592}\times \text{Radiomic signature}\\ \;+1.5085\times IF\left(minimum\;ADC=1\right)\\ \;+0.5829\times IF\left(minimum\;ADC=2\right)\\ \;+1.0957\times IF(MRI\text{-}reported\;N\;staging\\ \;=1)-1.5102 \end{array}$$

**Figure 4:**
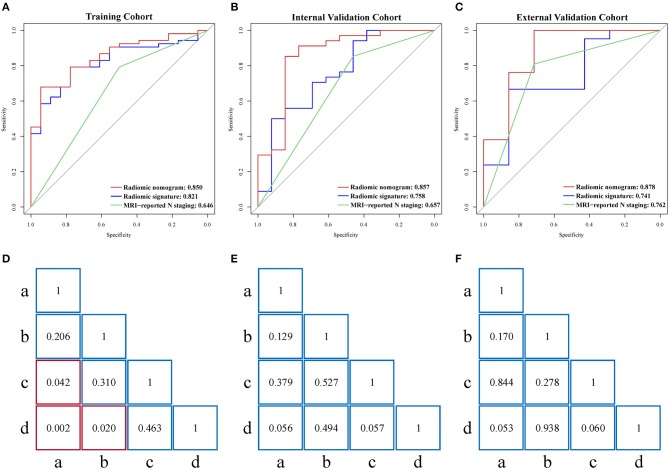
**(A–C)** Performance of radiomic nomogram, radiomic signature, and MRI-reported N staging scheme in the training, internal validation, and external validation cohorts. **(D–F)** Delong tests for AUCs of four models in the three cohorts. Red box represents *p* < 0.05. “a”, MRI-reported N staging; “b”, MRI-derived model; “c”, radiomic signature; “d”, radiomic nomogram; MRI, magnetic resonance imaging; AUC, area under the curve.

There was a significant correlation (Pearson's *r* = 0.530, 0.602, and 0.677) between radiomic nomogram and LNM status in the three cohorts. ROC curves are given in Figures 4A–C. Sensitivity, specificity, and accuracy results are presented in Table 2. In the internal validation cohort, our radiomic nomogram showed good discrimination performance of LNM status and surpassed the routinely used MRI-reported N staging, reaching an AUC of 0.857 vs. 0.657, with an accuracy of 0.851 vs. 0.745, a specificity of 0.846 vs. 0.462, and a same sensitivity of 0.853. Compared with the MRI-derived model, our radiomic nomogram still showed superior predictive ability with an AUC of 0.857 vs. 0.818, an accuracy of 0.851 vs. 0.702, and a sensitivity of 0.853 vs. 0.618, although falling behind a little in specificity. In the external validation cohort, the radiomic nomogram also outperformed MRI-reported N staging in AUC (0.878 vs. 0.762), sensitivity (0.952 vs. 0.810), and accuracy (0.878 vs. 0.786). Similarly, the radiomic nomogram could still compensate the MRI-derived model for sensitivity and accuracy. Figures 4D–F show the quantitative AUC comparisons of the four models with Delong test.

**Figure 5:**
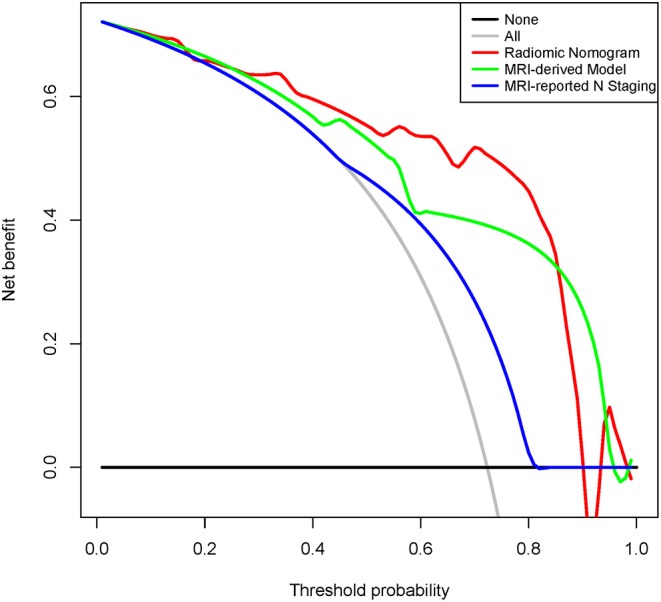
Decision curve analysis for radiomic nomogram, MRI-derived model, and MRI-reported N staging in the internal validation cohort. Red line represents radiomic nomogram. Green line represents MRI-derived model. Blue line represents MRI-reported N staging. Gray line assumes all patients have LNM. Black line assumes no patients have LNM. MRI, magnetic resonance imaging; ADC, apparent diffusion coefficient; LNM, lymph node metastasis.

As shown in Supplementary Figure 4, calibration curves of the radiomic nomogram suggested an agreement between model and actual outputs. Furthermore, DCA (Figure 5) indicated that the radiomic nomogram added more benefit when directing treatment decisions if the threshold probability was set between 0.24 and 0.86 compared with treat-none, treat-all, MRI-derived model, and MRI-reported N staging scheme.

## Discussion

In this study, we established a radiomic nomogram which incorporated the radiomic signature and clinical factors including the minimum ADC value and MRI-reported N staging for non-invasive prediction of LNM in AGC patients. The radiomic nomogram showed better performance in determining and evaluating preoperative LNM status than clinical radiologists. The practical radiomic nomogram could facilitate a more accurate and objective assessment of LNM in AGC while providing personalized support for clinical decision making.

In terms of machine learning radiomics, typical LASSO method followed by OOB bootstrapping estimates of different feature subsets defined by LVQ was adopted to select crucial radiomic features in this study, which were later on fed to the generally used logistic regression for model building. Jiang's study (4) analyzed the association between computed tomography (CT)-based radiomic signature and LNM in gastric cancer using LASSO logistic regression. Taking a step forward, Wang's study (8) used ICC for feature selection and random forest algorithm to construct a radiomic signature. Upon the consistence in feature selection and model building with their studies, our radiomic models not only brought a novel view of LVQ in radiomics methods but also achieved similar model performance.

Radiomic features adopted in this study were both texture features about informational measure of correlation between local grayscale pixels calculated from gray level co-occurrence matrix. Results of cross-validation showed their great stability. Further analysis of these two features revealed that the radiomic signature score increased as the values of *square_glcm_Imc1* and *wavelet.LLH_glcm_Imc2* decreased according to the radiomic signature formula, which represented the uneven texture features of images and high heterogeneity of tumors. This suggested that radiomic signature could reflect a preclinical potential in establishing a connection between image information and LNM status.

LNM is an intricate biological process in AGC, in which the primary tumor lesions undoubtedly play an important role (14–16). Jiang's study (4) established a radiomic nomogram based on CT images and clinicopathological findings to estimate the LNM in patients with gastric cancer. However, the ROIs only covering the single maximum level of the tumor lesion may lead to incomplete radiomic features. Besides, some small lymph nodes have metastasized, while large lymph nodes may be simply caused by inflammation, so the judgment of CT-reported findings could also bring some bias. Compared to CT images, MRI signal variations are more visible to detect and diagnose qualitatively (17–19). However, cases need to be noted that nodes with a diameter <8 mm or no obvious signal changes were later found to be metastatic nodes, while the opposite were found benign (20, 21). The low specificity in radiological diagnosis of LNM would preferentially overestimate the severity of disease and lead to excessive medical treatment. Without taking sample bias into account, a possible explanation was that while the tumor cells had already invaded into lymph nodes, changes in morphology and MRI signal were unlikely to present during the incubation period (18–21). The results of the current study thus showed the predictive power and potential for radiomics to reveal additional information invisible to the naked eye.

The ADC value mainly reflects tumor cell signal as a functional index that may provide an effective approach for the judgment of malignancy clinically (18, 20). Previous studies (3, 6, 19) have qualitatively studied the ADC value on the target lymph nodes, revealing a great correlation between low ADC value and metastatic nodes. Liu's study (22) showed that LNM had a correlation with ADC values of gastric cancer tissue. Traditional cognition displayed a greater tendency for a target node with a lower ADC to have greater malignancy (18). However, previous research results did not mention whether it was the average or the minimum ADC value, so many articles adopted default average ADC values. However, tumor heterogeneity should not only be expressed by a simple average ADC value but also by minimum ADC values, which would reflect the most heterogeneous ingredient in pathology type. By studying subjects with a minimum ADC value <700 and 600 mm^2^/s in this dataset, 52.9% (9/17) and 100% (3/3) of the cases were pathologically confirmed as a negative LNM, respectively. A possible explanation for this result is that the tumor cells have high malignancy and increased cell alignment (23). Besides, unabsorbed hematoma may also lead to an extreme minimum ADC value. Even so, the nomogram indicated improved discrimination for nodal assessment with a reported accuracy of 85% compared to 75 and 50% for endoscopic ultrasound and CT, respectively (24). This was an innovative attempt in the image-data combination era in AGC, and further study would improve upon the construction and development of radiomic nomograms with increased sample sizes and upgraded iterations of technological computer-aided algorithms (25).

Considering the close relationship between T staging and the presence of peritoneal seeding (4, 17), we hypothesized a connection between LNM and T staging. However, the actual results showed no statistical significance (*p* = 0.1726). Some tumor indicators and combined markers have been shown to be associated with LNM in gastric cancer. However, the results showed no significant correlation in this study (*p* = 0.2458). An explanation for this phenomenon was that combined markers were only divided into negative and positive results. Some indicators only showed significance when they were many times higher than the normal value (26).

The quality of the VOI could directly affect final experimental results, as the VOI acquisition was the raw material of all procedures (9). DWI sequence was utilized, as the gastric lesions showed better contrast and clearer circumscription even if some lesions were in high-grade T staging or exhibited invasive growth (16). To avoid the influence of lymph node signals and visual judgment errors, each modality was necessary to combine multiple sequences as a reference to accurately judge the profile of lesions (14, 16, 17). The shape of gastric tumors was irregular in their appearance on cross-section due to the congenital differences in anatomical location and morphology (19). Hence, the VOI of AGC lesions may have some insufficiency in terms of volume- and shape-related radiomic signature.

Despite the advantages offered by the approaches presented herein, there are some limitations to be noted. The inherent selection bias and an incomplete dataset are known issues of retrospective studies. The robustness and reproducibility of the radiomic models, although validated in an external cohort, still need to be optimized using a larger sample size. Subsequent studies should subdivide the N staging into more categories, especially for N3a and N3b staging, which would be of great use for clinical decision making (16, 27).

In conclusion, this study provided a radiomic nomogram incorporating the radiomic signature, minimum ADC value, and MRI-reported N staging, to establish an effective method to improve the preoperative individualized predictive efficacy of LNM in AGC patients conveniently and accurately.

## Data Availability Statement

The datasets for this manuscript are not publicly available because of patient information privacy. Requests to access the datasets should be directed to corresponding author (MX), xums166@zcmu.edu.cn.

## Ethics Statement

The studies involving human participants were reviewed and approved by the First Affiliated Hospital of Zhejiang Chinese Medical University and Hangzhou Hospital of Traditional Chinese Medicine. Written informed consent for participation was not required for this study in accordance with the national legislation and the institutional requirements.

## Author Contributions

JT and MX conceived and launched this study. WC, SW, and DD designed the medical and statistical analyses. WC, KZ, and JL collected cases and acquired clinical information. XG and BL implemented the control of image quality and clinical diagnosis. SW and HL analyzed the data and carried out statistical experiments. SW, XW, and MF provided result interpretation. WC and SW wrote the first draft of this manuscript. DD, JT, and MX revised and edited the final version. XG, KZ, JL, BL, HL, XW, and MF reviewed the manuscript.

### Conflict of Interest

The authors declare that the research was conducted in the absence of any commercial or financial relationships that could be construed as a potential conflict of interest.

## Abbreviations

ADC: apparent diffusion coefficient
AGC: advanced gastric cancer
AUC: area under the curve
DCA: decision curve analysis
DWI: diffusion-weighted imaging
ICC: intraclass correlation coefficient
LASSO: least absolute shrinkage and selection operator
LNM: lymph node metastasis
LVQ: learning vector quantization
MRI: magnetic resonance imaging
OOB: out-of-bag
ROC: receiver operating characteristic
VOI: volume of interest.
