# Supplementary material for: Evaluation of Lymph Node Metastasis in Advanced Gastric Cancer Using Magnetic Resonance Imaging-Based Radiomics
Source: Front Oncol. 2019 Nov 22;9:1265. doi: 10.3389/fonc.2019.01265 (PMC6883384; doi:10.3389/fonc.2019.01265)
Supplement: Supplementary file 1 [file Table_1.DOCX]

Supplementary Material

# Supplementary Methods

## Radiomic Feature Extraction

Radiomic features were extracted from the cancerous tumor region of each patient based on algorithms provided in Pyradiomics (version 2.1.1), including three categories (Supplementary Table 2): shape features (n = 13, describing the three-dimensional size and shape of the volume of interest (VOI) and only calculated on the non-derived image and mask), first-order features (n = 262, describing the distribution of voxel intensities within the image region defined by the mask through commonly used and basic metrics) and texture features (n = 1030, calculated from gray level cooccurrence matrix (GLCM), gray level run length matrix (GLRLM), gray level size zone matrix (GLSZM), neighboring gray tone difference matrix (NGTDM) and gray level dependence matrix (GLDM)). Also, original images were mainly transformed by square filters and wavelet filters.

For intraclass, we used a single-rating, consistency, 2-way mixed-effects model. For interclass, we chose a multiple-rating, consistency, 2-way random-effects model. In general, 813 radiomic features with intraclass correlation coefficients (ICCs) greater than 0.75 were extracted for radiomic signature building. ICCs for intraclass ranged from 0.751 to 1.000 and the inter-observer ICCs ranged from 0.752 to 1.000, indicating favorable reproducibility.

## Statistical Analysis Packages

R packages used in this study are as following. ICCs were calculated based on ‘psych’ package (version 1.7.8). The least absolute shrinkage and selection operator (LASSO) regression was carried out by use of package ‘glmnet’ (version 2.0-13). Learning vector quantization (LVQ) method was performed with package ‘mlbench’ (version 2.1-1) and ‘caret’ (version 6.0-81). The multivariate logistic regression for radiomic signature and nomogram construction were performed using the ‘rms’ package (version 5.1-2). The receiver operating characteristic (ROC) curves were conducted by ‘pROC’ package (version 1.10.0). The confidence intervals for sensitivity, specificity, and accuracy were calculated by ‘reportROC’ package (version 3.2).

## Explanations for Two Selected Radiomic Features

Selected features are both texture features calculated from GLCM, assessing the correlation between the probability distributions of $i$ and $j$ (quantifying the complexity of the texture). Detailed information are as follows.

1. *square_glcm_Imc1* is a texture feature calculated from GLCM using mutual information. Square filter takes the square of the image intensities and linearly scales them back to the original range. Then negative values in original images will be made negative again.

$$Imc1=\frac{HXY-HXY1}{max\left\{ HX,HY \right\}}$$

2. *wavelet.LLH_glcm_Imc2* is a texture feature calculated from GLCM based on the wavelet decomposition of the original image. Wavelet filters yield eight decompositions per level applying the combinations of either a High or a Low pass filter in each of the three dimensions.

$$Imc2=\sqrt{1-e^{-2\left( HXY2-HXY \right)}}$$

where

- $HX=-\sum_{i=1}^{N_{g}} p_{x}\left( i \right){log}_{2} \left( p_{x}\left( i \right)+\epsilon\right)$ be the entropy of $p_{x}$
- $HY=-\sum_{j=1}^{N_{g}} p_{y}\left( j \right){log}_{2} \left( p_{y}\left( j \right)+\epsilon\right)$ be the entropy of $p_{y}$
- $HXY=-\sum_{i=1}^{N_{g}} \sum_{j=1}^{N_{g}} p\left( i,j \right){log}_{2} \left( p\left( i,j \right)+\epsilon\right)$ be the entropy of $p\left( i,j \right)$


$HXY1=-\sum_{i=1}^{N_{g}} \sum_{j=1}^{N_{g}} p\left( i,j \right){log}_{2} \left( p_{x}\left( i \right)p_{y}\left( j \right)+\epsilon\right)$

$HXY2=-\sum_{i=1}^{N_{g}} \sum_{j=1}^{N_{g}} p_{x}\left( i \right)p_{y}\left( j \right){log}_{2} \left( p_{x}\left( i \right)p_{y}\left( j \right)+\epsilon\right)$

- $N_{g}$ be the number of discrete intensity levels in the image
- $P_{x}\left( i \right)$ be the marginal row probabilities and $P_{y}\left( j \right)$ the marginal column probabilities
- $p\left( i,j \right)$ be the normalized co-occurrence matrix
- $\epsilon$ be an arbitrarily small positive number ($\approx2.2\times{10}^{-16}$)

# Supplementary Figures and Tables

## Supplementary Figures


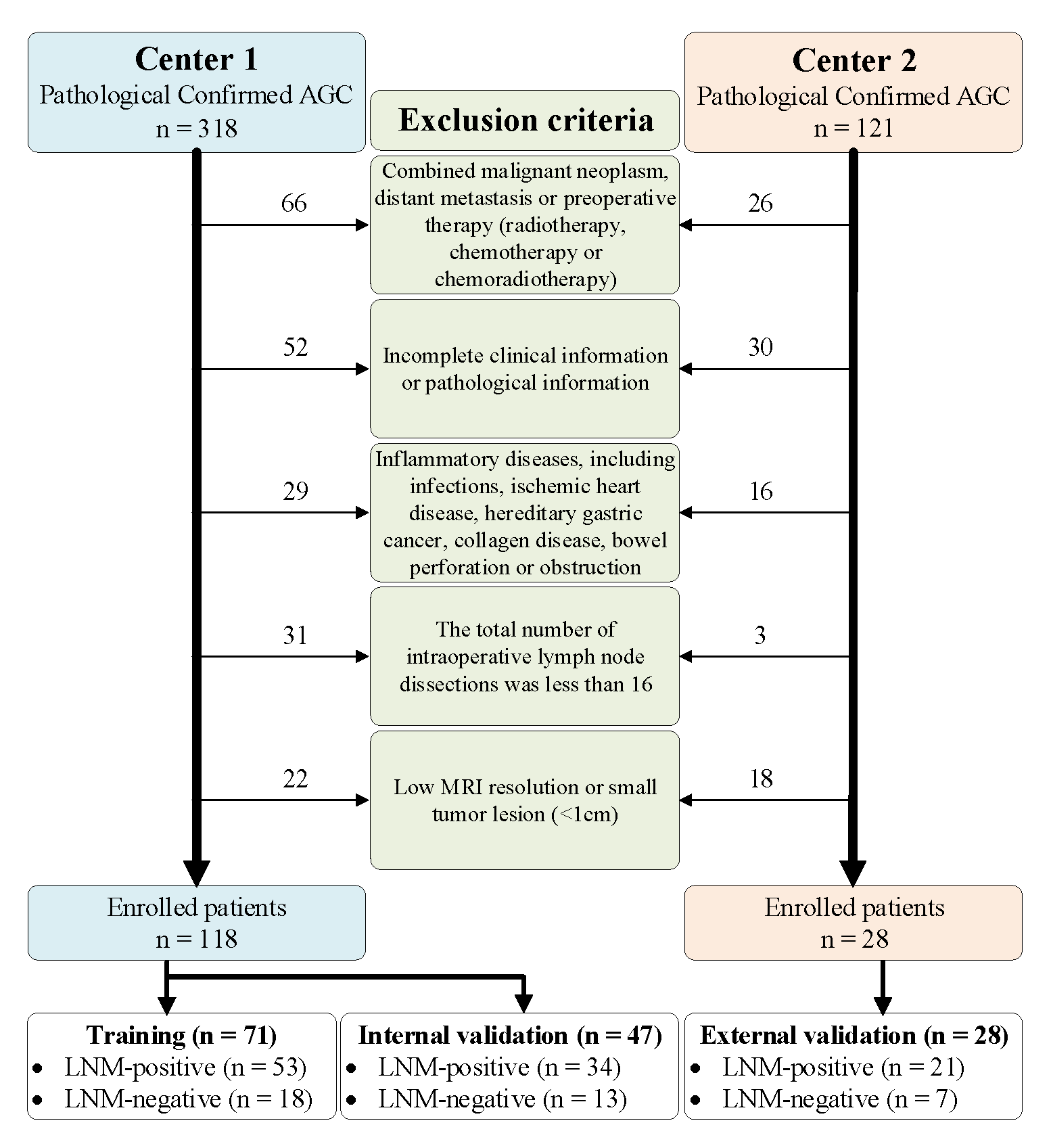


**Supplementary Figure 1.** Recruitment diagram for study population. Center 1 included patients from the First Affiliated Hospital of Zhejiang Chinese Medical University. Center 2 included patients from Hangzhou Hospital of Traditional Chinese Medicine. AGC, advanced gastric cancer; LNM, lymph node metastasis.


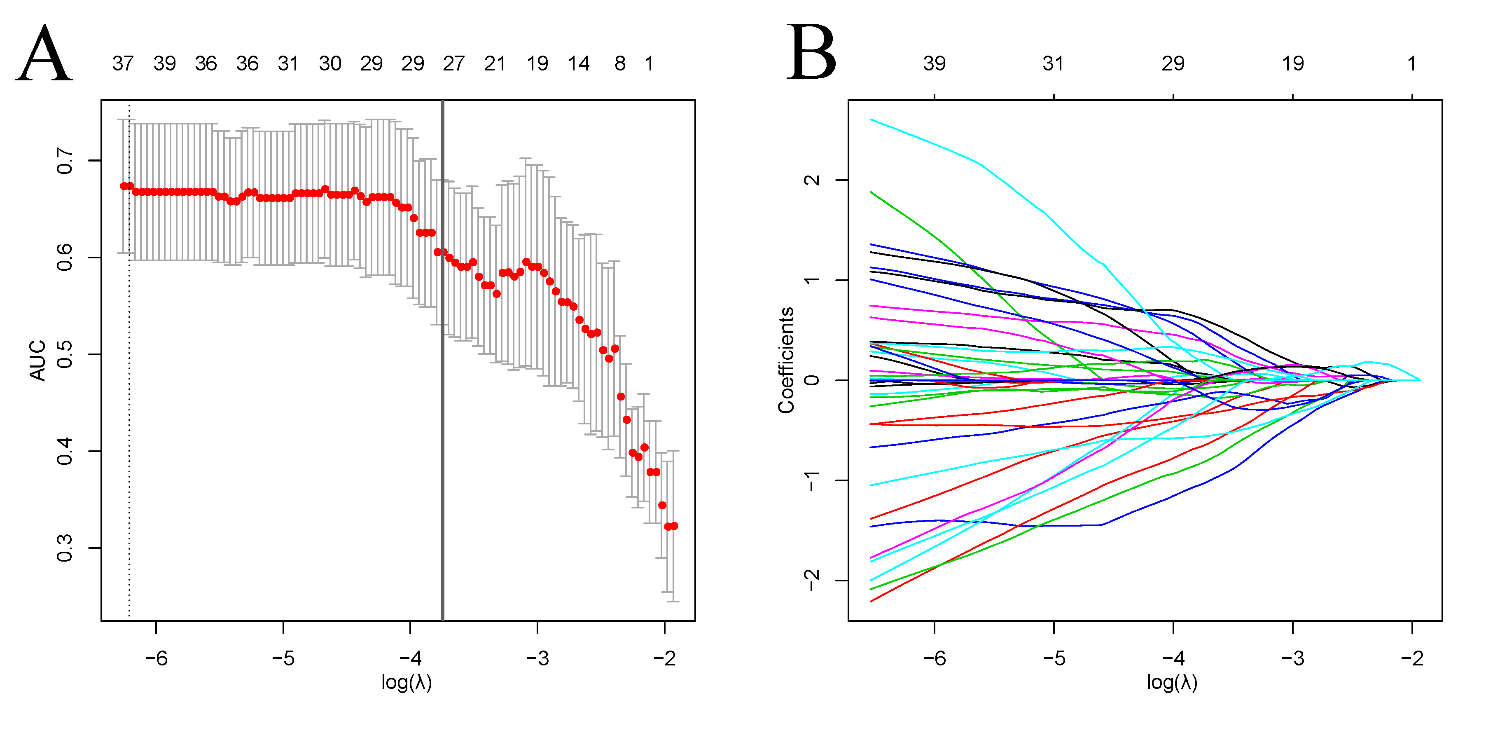


**Supplementary Figure 2.** (A) The tuning parameter ($\lambda$) selection in the LASSO model using 5-fold cross-validation via 1 standard error of the minimum criteria (1-SE criteria). The area under the receiver operating characteristic curve (AUC) was plotted versus log ($\lambda$). The dotted vertical line represents the optimal values by 1-SE criteria, giving 29 potential features. (B) The coefficient profile plot, shrinking the coefficients of features to zero to select key features. LASSO, least absolute shrinkage and selection operator.


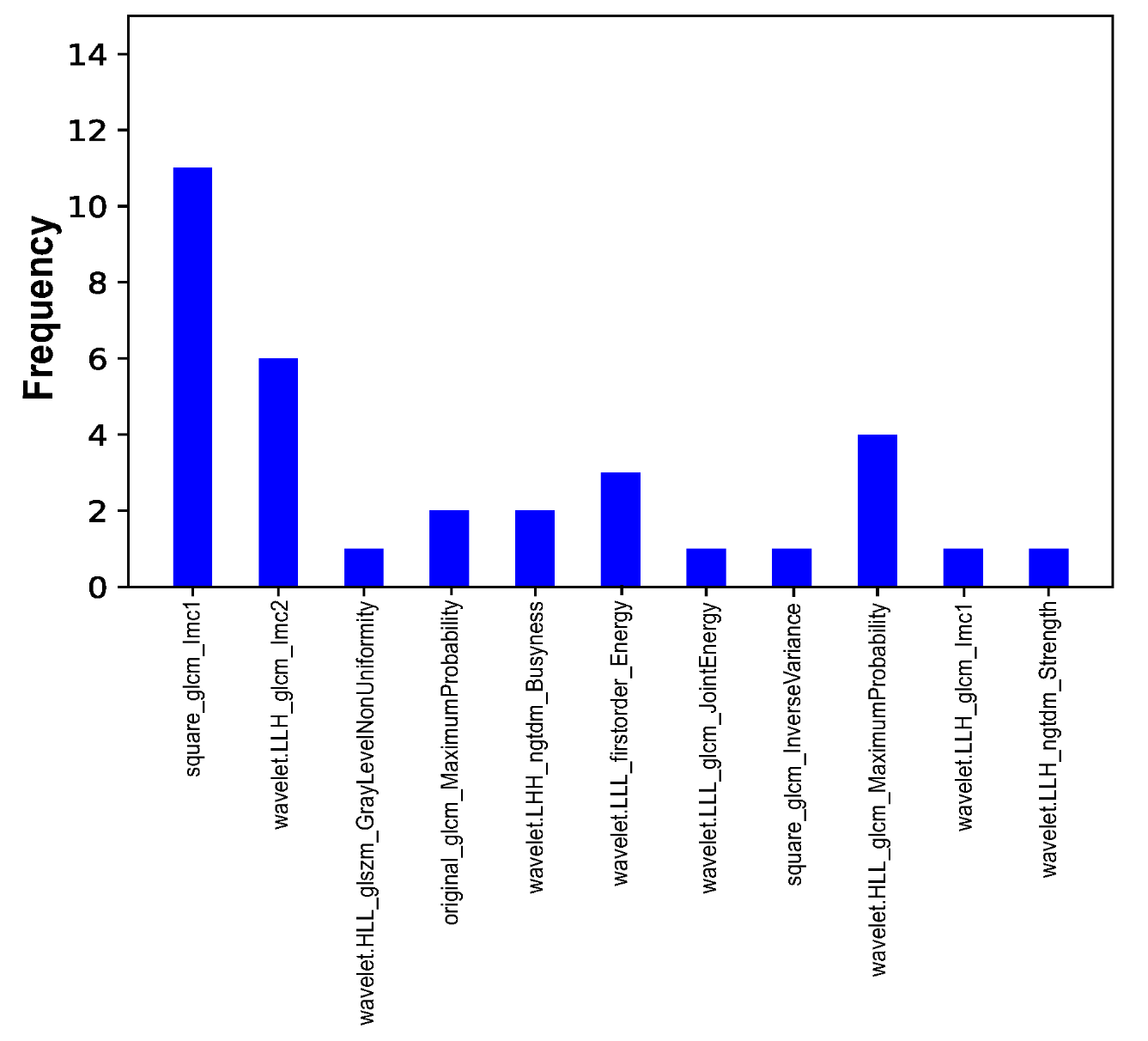


**Supplementary Figure 3.** Summarized frequency of features selected out in each fold of 10-fold cross-validation in the center 1 cohort. The x-axis represents eleven features from left to right respectively. The top two features are still the ones used in our radiomic signature.


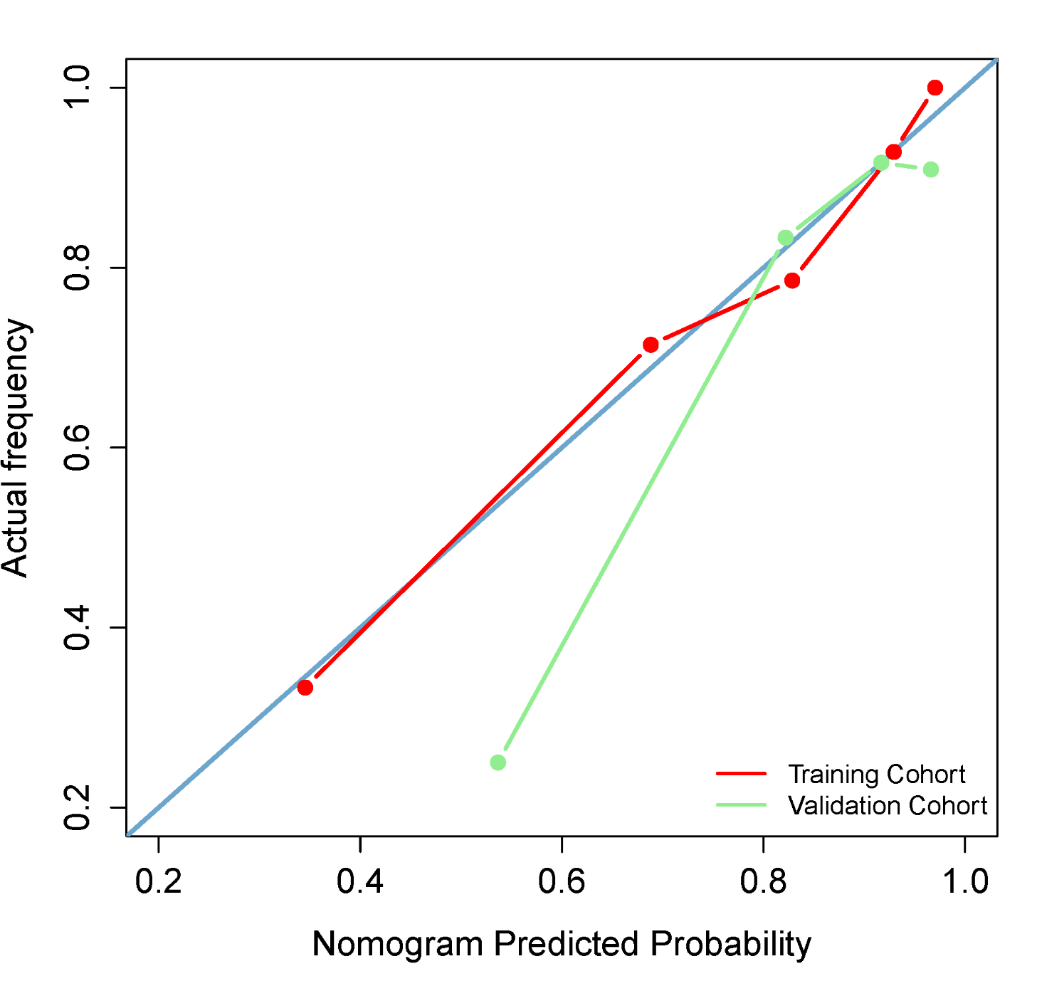


**Supplementary Figure 4.** Calibration curves of radiomic nomogram in the training cohort and internal validation cohort on discriminating LNM vs. non-LNM with *p* values of 0.956 and 0.061 (Chi-square test), respectively.

## Supplementary Tables

**Supplementary Table 1.** Clinical and imaging characteristics of patients with AGC in external validation cohort.

| Clinical factors | External Validation Cohort (n=28) | |
| --- | --- | --- |
|  | LNM (+) | LNM (-) |
| Age, mean ± SD, years | 60.57±12.99 | 64.14±17.23 |
| Sex, No. (%) |  |  |
| male | 14 (66.7) | 5 (71.4) |
| female | 7 (33.3) | 2 (28.6) |
| MRI-reported N staging, No. (%) |  |  |
| positive | 17 (81.0) | 2 (28.6) |
| negative | 4 (19.0) | 5 (71.4) |
| Average ADC value, mean, No. (%) | 1235 | 1538 |
| Minimum ADC value, No. (%) |  |  |
| 0 (<700) | 2 (9.5) | 0 (0.0) |
| 1 (700-1200) | 17 (81.0) | 1 (14.3) |
| 2($\boldsymbol{\geq}$1200) | 2 (9.5) | 6 (85.7) |
| Radiomic signature |  |  |
| median  (interquartile range) | 2.547  (1.868-3.165) | 1.883  (0.803-1.941) |
| Radiomic nomogram |  |  |
| median  (interquartile range) | 2.747  (2.500-3.355) | 0.691  (-0.237-1.288) |

NOTE. AGC, advanced gastric cancer; LNM, lymph node metastasis; MRI, magnetic resonance imaging; ADC, apparent diffusion coefficient; SD, standard deviation.

**Supplementary Table 2.** Extracted radiomic features.

| Matrix | Index |
| --- | --- |
| Shape features (n=13) | Volume |
|  | Surface Area |
|  | Surface Area to Volume ratio |
|  | Sphericity |
|  | Maximum 3D diameter |
|  | Maximum 2D diameter Slice |
|  | Maximum 2D diameter Column |
|  | Maximum 2D diameter Row |
|  | Major Axis Length |
|  | Minor Axis Length |
|  | Least Axis Length |
|  | Elongation |
|  | Flatness |
| First-order features (n=262) | Energy (n=15) |
|  | Total Energy (n=15) |
|  | Entropy (n=13) |
|  | Minimum (n=14) |
|  | 10th Percentile (n=15) |
|  | 90th Percentile (n=15) |
|  | Maximum (n=14) |
|  | Mean (n=15) |
|  | Median (n=14) |
|  | Interquartile Range (n=15) |
|  | Range (n=14) |
|  | Mean Absolute Deviation (n=15) |
|  | Robust Mean Absolute Deviation (n=15) |
|  | Root Mean Squared (n=15) |
|  | Skewness (n=15) |
|  | Kurtosis (n=15) |
|  | Variance (n=15) |
|  | Uniformity (n=13) |
| Texture features (n=1030) | Gray Level Co-occurrence Matrix (GLCM, n=299) |
|  | Gray Level Run Length Matrix (GLRLM, n=232) |
|  | Gray Level Size Zone Matrix (GLSZM, n=230) |
|  | Neighboring Gray Tone Difference Matrix (NGTDM, n=65) |
|  | Gray Level Dependence Matrix (GLDM, n=204) |

**Supplementary Table 3.** 95% confidence intervals for sensitivity, specificity, and accuracy.

| Cohorts | Models | Sensitivity (95% CI) | Specificity (95% CI) | Accuracy (95% CI) |
| --- | --- | --- | --- | --- |
| **Training** |  |  |  |  |
|  | MRI-reported N staging | 0.792 (0.683-0.902) | 0.500 (0.269-0.731) | 0.718 (0.713-0.724) |
|  | MRI-derived model | 0.528 (0.394-0.663) | 0.833 (0.661-1.000) | 0.606 (0.599-0.612) |
|  | Radiomic signature | 0.792 (0.683-0.902) | 0.778 (0.586-0.970) | 0.789 (0.784-0.793) |
|  | Radiomic nomogram | 0.679 (0.554-0.805) | 0.944 (0.839-1.000) | 0.746 (0.741-0.752) |
| **Internal validation** |  |  |  |  |
|  | MRI-reported N staging | 0.853 (0.734-0.972) | 0.462 (0.191-0.733) | 0.745 (0.737-0.753) |
|  | MRI-derived model | 0.618 (0.454-0.781) | 0.923 (0.778-1.000) | 0.702 (0.693-0.711) |
|  | Radiomic signature | 0.765 (0.622-0.907) | 0.462 (0.191-0.733) | 0.681 (0.672-0.690) |
|  | Radiomic nomogram | 0.853 (0.734-0.972) | 0.846 (0.650-1.000) | 0.851 (0.846-0.856) |
| **External validation** |  |  |  |  |
|  | MRI-reported N staging | 0.810 (0.642-0.977) | 0.714 (0.380-1.000) | 0.786 (0.774-0.798) |
|  | MRI-derived model | 0.714 (0.521-0.908) | 1.000 (1.000-1.000) | 0.786 (0.774-0.798) |
|  | Radiomic signature | 0.952 (0.861-1.000) | 0.429 (0.861-1.000) | 0.821 (0.811-0.832) |
|  | Radiomic nomogram | 0.952 (0.861-1.000) | 0.714 (0.380-1.000) | 0.893 (0.886-0.900) |

NOTE. MRI, magnetic resonance imaging; CI, confidence interval.
